# Effect of local melatonin application on immediately placed implants in maxillary premolars (a biochemical and radiographic randomized controlled clinical trial)

**DOI:** 10.1186/s12903-026-09968-y

**Published:** 2026-10-17

**Authors:** Toka Moustafa Helal, Magda Mohamed Saleh, Dina M. N. Metawie

**Affiliations:** Department of Oral and Maxillofacial Surgery, Faculty of Dentistry, Alexandria University, Alexandria, Egypt

**Keywords:** Immediately placed implants, Melatonin, Osteoprotegerin, CBCT, Relative bone density, Bone volume, Marginal bone loss

## Abstract

**Background:**

Melatonin, a potent antioxidant and bone-regulating hormone, promotes osteoblastic activity and inhibits osteoclastic differentiation primarily through upregulation of osteoprotegerin (OPG) and suppression of the receptor activator of nuclear factor-kappa B ligand (RANKL) pathway. Given its dual osteogenic and antiresorptive effects, melatonin may enhance bone remodelling around immediately placed implants. This study aimed to evaluate the effect of locally applied melatonin on peri-implant bone remodelling by assessing changes in OPG levels and radiographic bone parameters over time.

**Patients and methods:**

Twenty-four patients with non-restorable maxillary premolars were divided into two groups. The melatonin group received immediately placed implants with local melatonin application, and the control group received immediately placed implants without melatonin application. Serum OPG levels were measured one and three months postoperatively, and cone-beam computed tomography (CBCT) was performed immediately after implant placement and at six months postoperatively to assess relative bone density, bone volume, and marginal bone loss.

**Results:**

Biochemical findings: At one month, OPG levels were significantly higher in the melatonin group compared to the control group (*p* = 0.012). By 3 months, there was no statistically significant difference between the two groups (*p* = 0.707).

Radiographic findings: After six months, relative bone density was numerically higher in the melatonin group than in the control group, although the between-group difference was not statistically significant (*p* = 0.372). However, the percentage increase from baseline was significantly greater in the melatonin group (*p* = 0.002). Bone volume decreased in both groups, with a percent reduction in bone volume that was significantly smaller in the melatonin group compared to the control group (*p* < 0.001). Marginal bone loss was significantly lower in the melatonin group compared to the control group (*p* < 0.001).

**Conclusion:**

Locally applied melatonin was associated with favourable early biochemical and radiographic outcomes, including a greater percentage increase in relative bone density from baseline, preservation of peri-implant bone volume, and reduced marginal bone loss.

**Trial registration:**

The trial was retrospectively registered at the Clinicaltrials.gov registry (Registration ID #NCT07103564), first submitted on July 29, 2025 https://clinicaltrials.gov/study/NCT07103564.

**Supplementary Information:**

The online version contains supplementary material available at https://doi.org/10.1186/s12903-026-09968-y.

## Introduction

Immediate implant placement has become a well-established treatment modality in implant dentistry because it shortens treatment time, preserves the alveolar socket architecture, particularly the buccal bone plate, and reduces the number of surgical interventions while maintaining favourable esthetic and functional outcomes in appropriately selected cases [1, 2]. However, successful peri-implant bone healing and remodelling remain critical determinants of treatment success, creating continued interest in adjunctive strategies that may enhance these biological processes.

Melatonin (N-acetyl-5-methoxytryptamine) is a neurohormone primarily secreted by the pineal gland and synthesized in several peripheral tissues, including the retina, skin, gastrointestinal tract, and bone marrow [3, 4]. Beyond its physiological role in regulating circadian rhythms, melatonin exhibits potent antioxidant, anti-inflammatory, and osteogenic properties [5]. It reduces oxidative stress, modulates inflammatory mediators, stimulates osteoblast proliferation and differentiation, enhances type I collagen synthesis, and suppresses osteoclastogenesis through the regulation of the receptor activator of nuclear factor-kappa B ligand (RANKL)/osteoprotegerin (OPG) signalling pathway, thereby promoting bone formation and limiting bone resorption [5, 6].

Bone remodelling around dental implants is largely regulated by the RANK/RANKL/OPG signalling pathway [7]. OPG, a soluble decoy receptor for RANKL, inhibits osteoclast differentiation and activity, thereby maintaining the balance between bone formation and bone resorption [8]. Since melatonin has been shown to increase OPG expression while suppressing RANKL, monitoring serum OPG levels may serve as a valuable biochemical indicator of peri-implant bone remodelling and early osseointegration following local melatonin application [9, 10].

Experimental and animal studies have consistently demonstrated the beneficial effects of melatonin on bone regeneration and osseointegration, including enhanced osteoblast activity, increased bone-to-implant contact, improved new bone formation, and accelerated peri-implant healing [11, 12].

Recent systematic reviews have further highlighted the therapeutic potential of melatonin as an adjunctive agent in dental and implant-related procedures, while emphasizing that high-quality clinical evidence remains limited [13–16].

In addition, recent randomized clinical trials have further expanded the potential applications of locally delivered melatonin in oral regenerative procedures. Adjunctive local melatonin has also demonstrated favourable clinical, radiographic, and histomorphometric outcomes in maxillary sinus augmentation [17]. Additionally, its use as a local drug delivery agent in periodontal therapy has shown significant improvements in clinical and inflammatory parameters. These findings further support the biological rationale for the local use of melatonin to enhance oral hard and soft tissue healing [18, 19].

Nevertheless, despite these promising findings, the translation of this evidence into clinical implant practice remains limited. Most published studies have been experimental or animal investigations, whereas well-designed randomized clinical trials evaluating the local application of melatonin on immediately placed dental implants are still scarce, particularly those integrating both biochemical and radiographic outcome measures.

To the best of our knowledge, no previous randomized controlled clinical trial has investigated the effect of locally applied melatonin on immediately placed implants by combining serum OPG assessment with radiographic evaluation of relative bone density, peri-implant bone volume, and marginal bone loss. Accordingly, the present randomized controlled clinical trial was designed to address this important knowledge gap by providing a biochemical and radiographic evaluation of the effect of locally applied melatonin on immediately placed dental implants. The null hypothesis was that local application of melatonin around immediately placed implants would not result in significant differences in biochemical or radiographic parameters of peri-implant bone healing compared with the control group. The primary outcome of this randomized controlled clinical trial was to evaluate the effect of locally applied melatonin around immediately placed implants in maxillary premolars on peri-implant relative bone density, assessed using standardized CBCT imaging immediately after implant placement and at the six-month postoperative follow-up. Secondary outcomes included changes in serum osteoprotegerin levels at one and three months postoperatively, as well as peri-implant bone volume and marginal bone loss, assessed using the same standardized CBCT protocol at both imaging time points.

## Materials and methods

### Ethical approval and informed consent

This randomized controlled clinical trial (1:1 ratio) followed CONSORT guidelines [20]. Participants were recruited from the Oral and Maxillofacial Surgery Department, Faculty of Dentistry, Alexandria University (July 2024–July 2025). The study was retrospectively registered at ClinicalTrials.gov (NCT07103564) and approved by the Faculty Ethics Committee (IORG0008839; No. 0812 − 12/2023), following the Declaration of Helsinki [21]. Before the intervention, written informed consent was provided by participants after they had been thoroughly informed about the study’s methods.

### Sample size calculation

The sample size was determined using an 80% research power and a 5% alpha error. The mean ± SD of relative bone density was 692 ± 165.65 in the Melatonin group and 539.45 ± 84.7 in the control group [22]. The sample size was calculated using independent means using a pooled SD = 125.175; thus, a sample of 11 per group was required. This was increased to 12 samples to make up for cases lost to follow-up. Total sample = Number per group x Number of groups = 12 × 2 = 24 patients.

### Software

The sample size was determined using Brant’s sample size calculator at the University of British Columbia, based on Rosner’s [23] technique [24] .

### Patient selection

Twenty-four patients presenting with non-restorable maxillary premolars who fulfilled the study eligibility criteria were recruited from the outpatient clinic of the Department of Oral and Maxillofacial Surgery, Faculty of Dentistry, Alexandria University.

#### Inclusion criteria

Patients aged 25–50 years presenting with non-restorable maxillary premolars indicated for immediate implant placement were included. Eligible sites demonstrated favourable clinical and radiographic conditions for immediate implant placement without the need for simultaneous guided bone regeneration (GBR), as determined by the preoperative clinical examination and CBCT. Participants had no local or systemic conditions that could adversely affect bone healing [25], were non-smokers [25], and demonstrated good compliance with follow-up visits [26].

#### Exclusion criteria

Patients with uncontrolled systemic diseases affecting healing (e.g., uncontrolled diabetes mellitus) [25], periapical pathology contraindicating immediate implant placement [26], a history of head and neck irradiation [26], or a history of using medications known to affect bone metabolism (e.g., bisphosphonates, corticosteroids, denosumab, or other anti-resorptive or anabolic bone agents) were excluded.

### Randomization and blinding

Participants were randomly allocated into the study and control groups using a computer-generated permuted block randomization sequence with a block size of four generated through the Sealed Envelope™ Simple Randomization Service [27]. The randomization sequence was generated by a trial-independent individual who was not involved in participant recruitment, treatment, or outcome assessment. Allocation concealment was ensured using sequentially numbered, opaque, sealed envelopes, which were prepared and kept by the same independent individual and opened only after participant enrolment, immediately before surgery. Participant screening, eligibility assessment, and enrolment were performed by the study supervisor, while all surgical procedures were performed by the principal investigator. Blinding of the operator and participants was not feasible because of the nature of the intervention. However, all CBCT measurements were performed by a blinded radiographic examiner who was unaware of the treatment allocation throughout the image analysis.

### Interventions

**Fig. 1:**
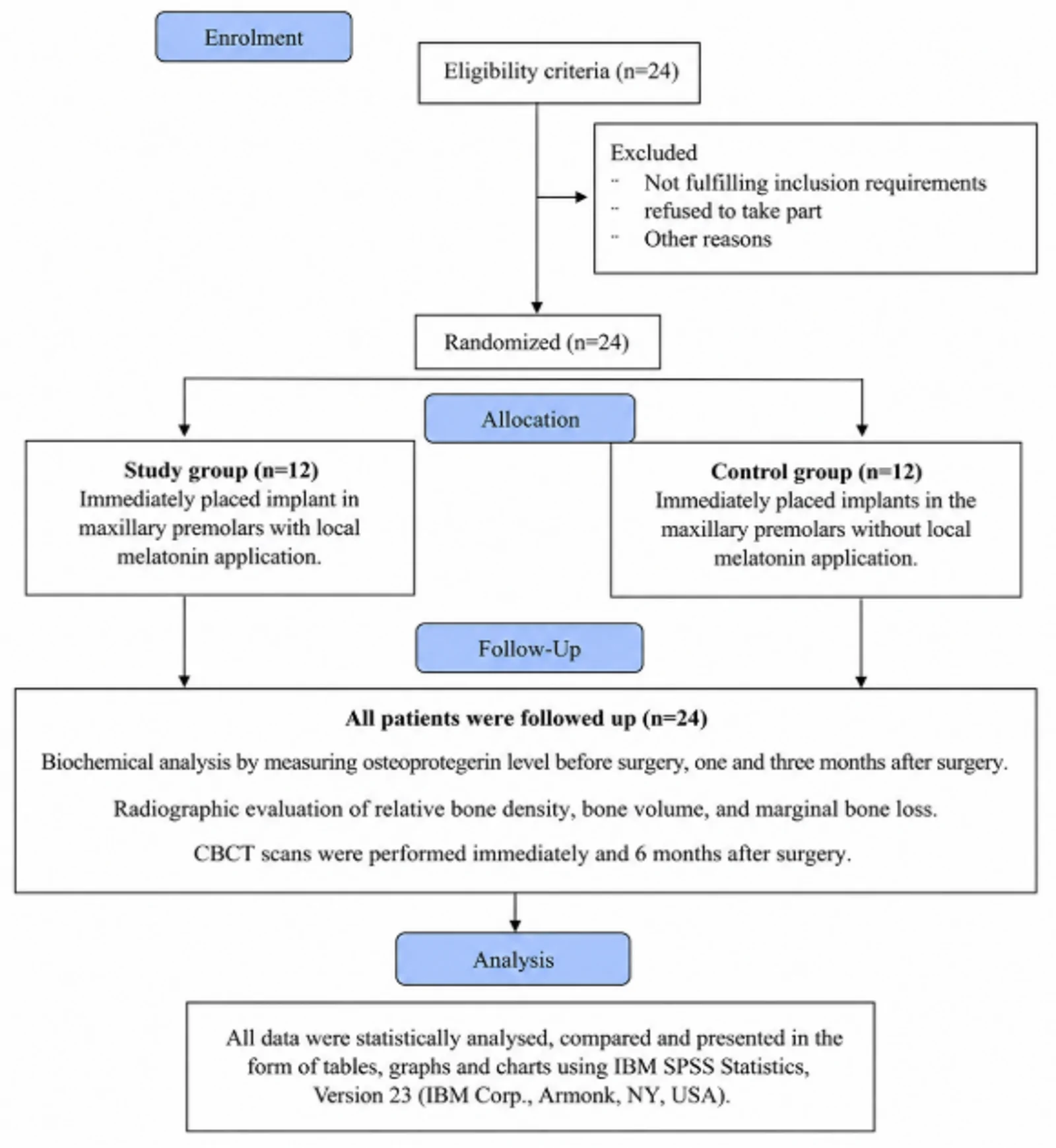
Flow chart illustrating trial participants’ development in accordance with CONSORT guidelines

The study group consisted of twelve patients who received implants immediately after atraumatic maxillary premolars extraction with local melatonin application. The control group consisted of twelve patients who received immediately placed implants after atraumatic extraction of maxillary premolars without local melatonin application (Fig. 1).

#### Preoperative phase

The patient’s primary complaint, as well as personal, prior dental, and medical records, were included in the preoperative evaluation. A thorough clinical examination was performed to ensure there was no suppuration, discharge, or swelling. A preoperative CBCT examination was performed for all participants as part of routine treatment planning to assess extraction socket morphology, evaluate the integrity of the surrounding alveolar bone, confirm the suitability of the site for immediate implant placement, and determine the appropriate implant dimensions. Only extraction sockets considered clinically suitable for immediate implant placement, with an intact buccal bone plate and favourable socket morphology that did not require simultaneous bone augmentation, were included according to the study eligibility criteria.

#### Preoperative preparation and biochemical assessment

**Fig. 2:**
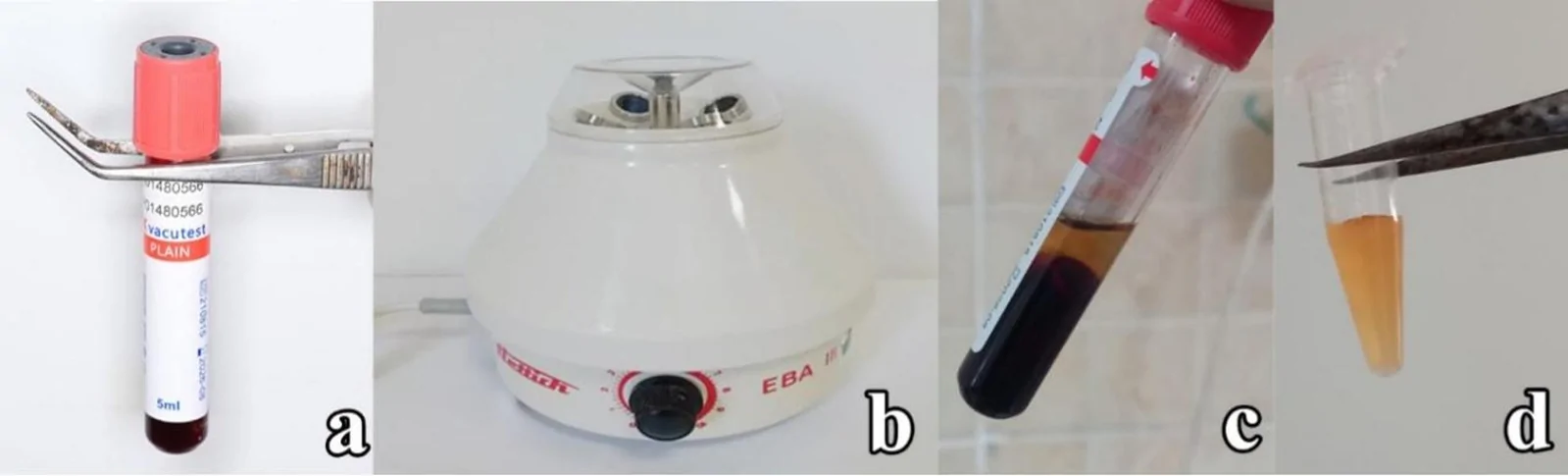
**A** The blood was drawn in a red top blood tube. **B** Hettich EBA III Laboratory Centrifuge − 4 × 15 / 20 ml − 6000 rpm. **C** The blood after centrifugation. **D** The separated serum is in an Eppendorf

All patients received oral hygiene instructions and underwent preoperative scaling and root planning before the surgical procedure. For biochemical evaluation, 5 mL of venous blood was collected before surgery to establish baseline OPG levels. Additional blood samples were collected at one and three months following implant placement to assess OPG levels. After allowing each sample to clot at room temperature, it was centrifuged for 15 min at 1000 g. The separated serum was stored at − 20 °C until further analysis (Fig. 2).

#### ELISA test principle

**Fig. 3:**
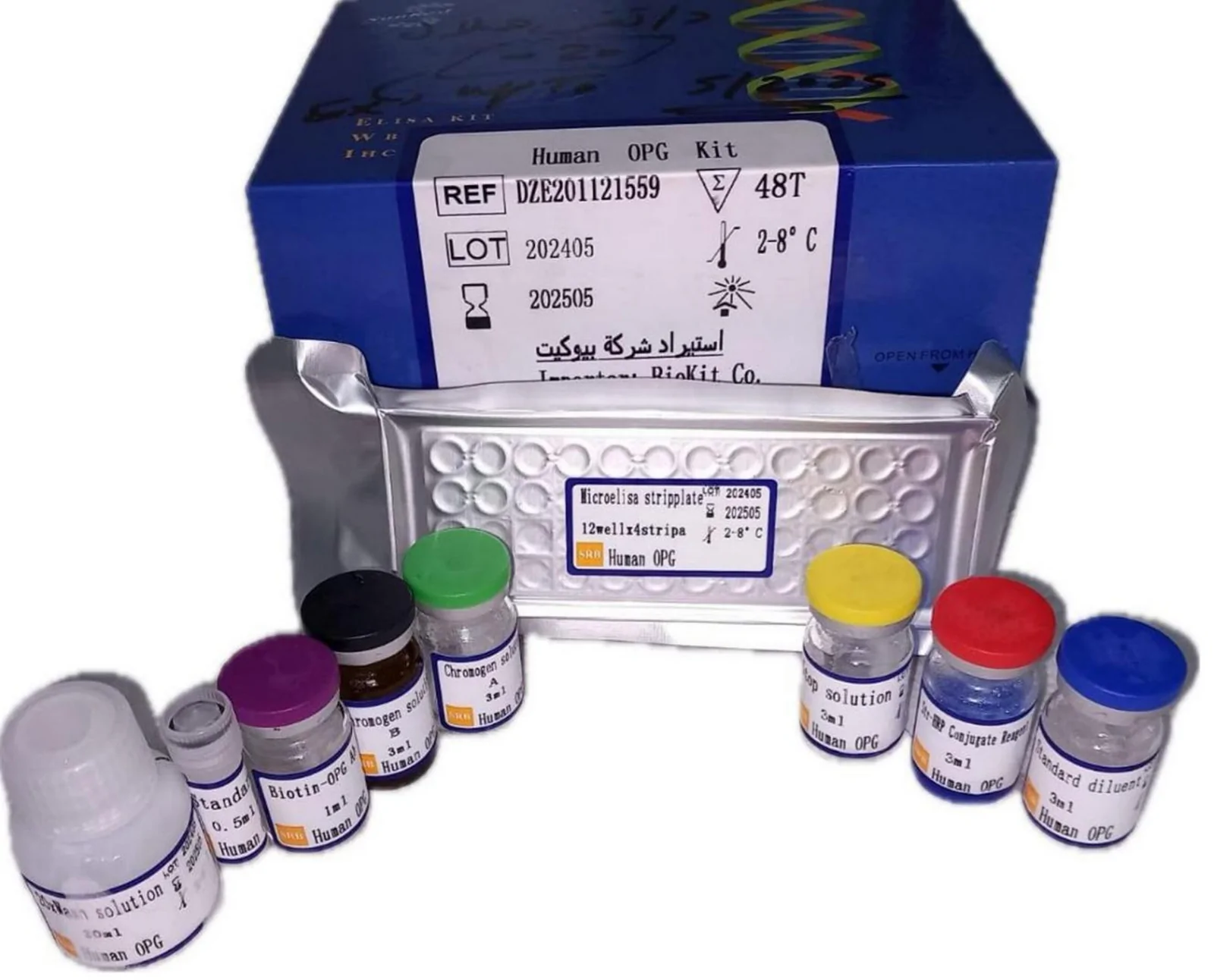
OPG ELISA kit (Catalogue No. 201-12-1559) Human Osteoprotegerin (OPG/TNFRSF11B) ELISA Kit (48T)

The double-antibody sandwich enzyme-linked immunosorbent assay (ELISA) method was used to measure OPG concentrations. In this method, serum samples were added to wells pre-coated with OPG-specific monoclonal antibodies and subsequently incubated with biotin-labelled detection antibodies and streptavidin–horseradish peroxidase (HRP) to form an immune complex. Following incubation and washing, chromogen solutions A and B were added to develop a yellow colour directly proportional to the OPG concentration. The optical density was measured spectrophotometrically, and OPG levels were calculated based on the standard calibration curve (Fig. 3).

#### Operative phase

All patients were instructed to rinse with a 0.12% chlorhexidine gluconate mouthwash (e.g., Hexitol^®^, The Arab Drug Company, Cairo, Egypt) for two minutes before surgery.

All surgical procedures were performed by the same experienced oral and maxillofacial surgeon following a standardized surgical protocol to minimize operator-related variability.

Under local anaesthesia, the surgery was done using buccal and palatal infiltrations with 4% articaine hydrochloride and 1:100,000 epinephrine (Alexadricaine^®^, Alexandria Company for Pharmaceuticals, Egypt). In both groups, atraumatic extraction of non-restorable maxillary premolars was performed, followed by irrigation and curettage of the socket with normal saline to remove any debris before drilling for the implant. The horizontal gap between the implant surface and the socket wall was clinically evaluated during implant placement. Only cases presenting a jumping gap of less than 2 mm were included; therefore, no simultaneous guided bone regeneration procedures were required.

**Fig. 4:**
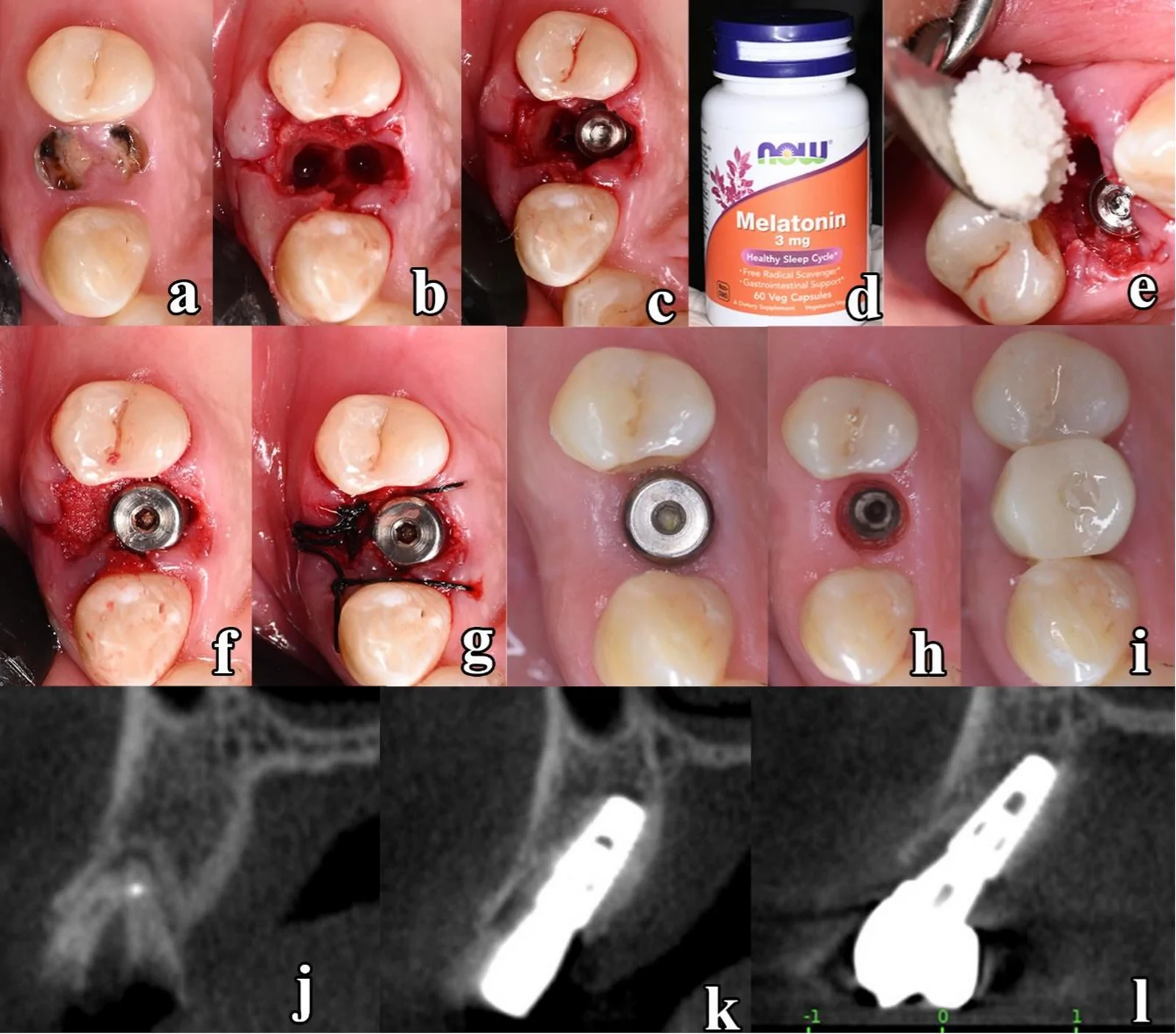
A case in the study group: (**A**) Preoperative photograph showing the remaining roots of the upper right first premolar. **B** Extraction socket after atraumatic extraction of the tooth. **C** The space visible after implant placement. **D** Melatonin powder (3 mg capsule; NOW Foods^®^, USA). **E** Application of the melatonin–saline mixture into the space. **F** The space filled with melatonin; a healing abutment was placed. **G** The wound was sutured with interrupted 4 − 0 silk sutures. **H** Soft tissue healing and suture removal after one week. **I** Final screw retained zirconia crown after six months. **J** Preoperative CBCT showing remaining roots. **K** CBCT immediately after implant placement. **L** CBCT after six months demonstrates bone remodelling

In the melatonin group, immediate implants were placed. All implants were placed according to a standardized surgical protocol using the Dentcon Dental Implant System (Dentimplant Medikal Sağlık, Ankara, Türkiye), manufactured from Grade IV titanium and featuring an 11° conical implant-abutment connection, an internal hex configuration, a self-cutting thread design, a rounded apex, and a hydrophilic SLA surface. Implant diameter and length were selected according to the available bone volume and extraction socket anatomy. Implants were positioned approximately 1–2 mm sub-crestal in all cases. After achieving primary stability with an insertion torque of 35–40 N·cm, the contents of a commercially available 3 mg melatonin capsule (NOW Foods^®^, USA), containing melatonin (N-acetyl-5-methoxytryptamine) as the active ingredient, were mixed with sterile saline immediately before application. The resulting melatonin–saline mixture was applied locally until complete filling of the peri-implant gap, and any remaining material was discarded (Fig. 4).

**Fig. 5:**
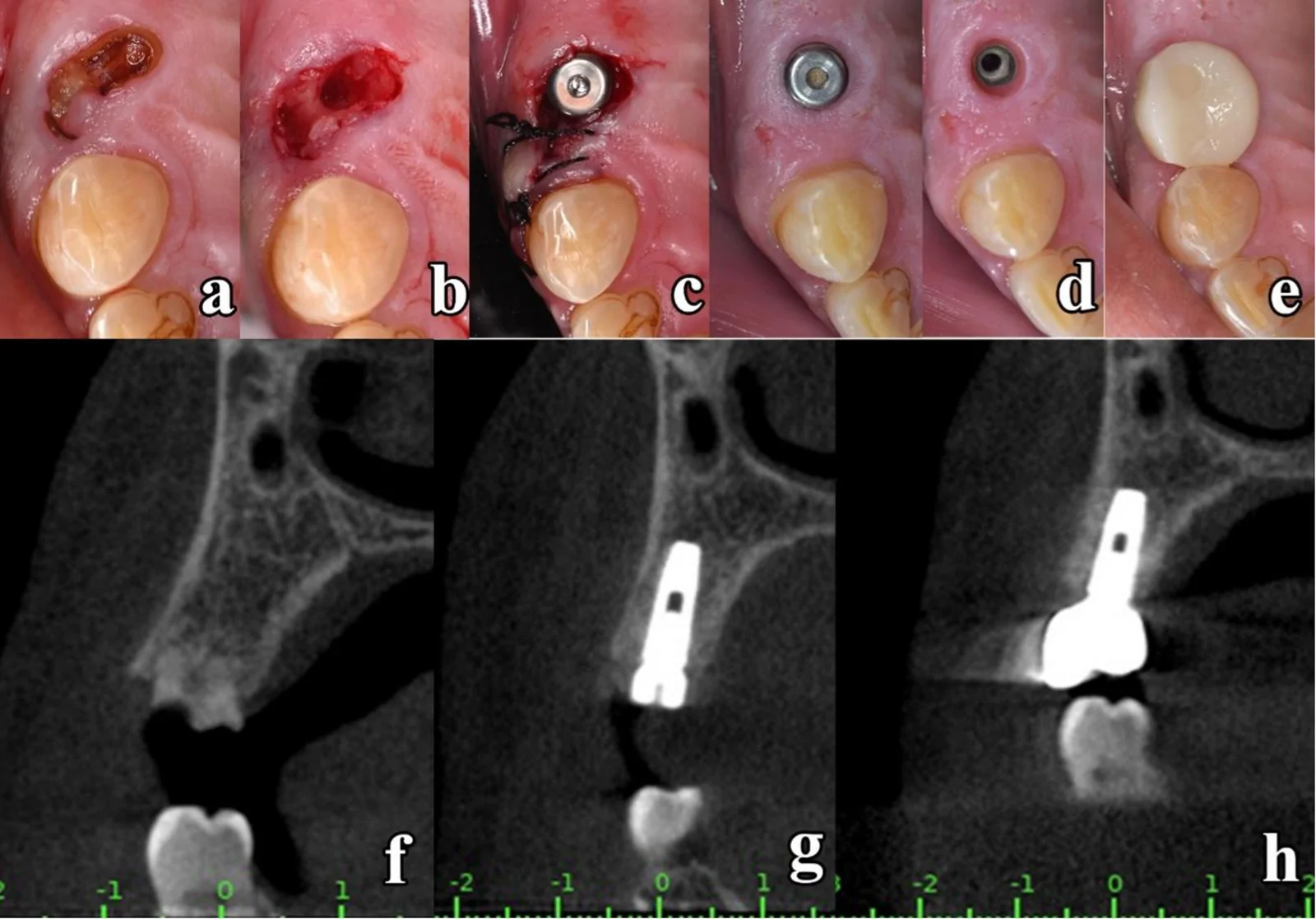
A case in the control group: (**A**) Preoperative photograph showing the remaining roots of the upper right first premolar. **B** Extraction socket after atraumatic removal of the tooth. **C** Wound sutured with interrupted 4 − 0 silk sutures. **D** Soft tissue healing and suture removal after one week. **E** Final screw retained zirconia crown placement after six months. **F** Preoperative CBCT showing the remaining roots. **G** CBCT immediately after implant placement. **H** CBCT after six months demonstrates peri-implant bone remodeling

In the control group, immediate implants were placed, achieving a primary stability with an insertion torque of 35–40 N· cm without melatonin application (Fig. 5).

In both groups, a healing abutment was attached to the implant, and the soft tissues were sutured using interrupted 4 − 0 silk sutures placed around the abutment.

#### Post-operative phase

All patients were prescribed amoxicillin–clavulanic acid (Augmentin^®^, 1 g, twice daily; GlaxoSmithKline, UK) and diclofenac potassium (Cataflam^®^, 50 mg, three times daily; Novartis, Switzerland) for five days following implant placement. Chlorhexidine gluconate mouthwash (Hexitol^®^, 0.12%; The Arab Drug Company, Cairo, Egypt) was used twice daily starting on the second postoperative day and continued for 10 days. Sutures were removed after 7–10 days.

### Biochemical and radiographic evaluation

#### Biochemical evaluation

Serum OPG levels (ng/mL) were assessed in blood samples collected preoperatively (baseline) and at 1 and 3 months after implant placement using a commercially available Human Osteoprotegerin (OPG/TNFRSF11B) ELISA kit [28] (Catalogue No. 201-12-1559; Sunlong Biotech Co., Ltd., Zhejiang, China) (Fig. 3). Serum OPG was selected as the predefined biochemical outcome measure to longitudinally monitor changes in circulating bone remodelling throughout the healing period, while radiographic analyses were performed to evaluate local peri-implant bone healing.

#### Postoperative radiographic evaluation

The same imaging parameters were used to acquire CBCT scans both immediately after implant placement (baseline) and at the six-month follow-up visit immediately before prosthetic loading. DICOM files were exported and analysed using Atomica™ AI software (version 3.0.1; Atomica AI, Atlanta, GA, USA).

##### Relative bone density

For each implant, standardized cross-sectional slices were examined perpendicular to the implant’s long axis. Two regions of interest of identical size were selected in the buccal and palatal peri-implant bone, positioned about 1 mm away from the implant surface to minimize the effect of artifacts and beam hardening.

The estimated gray value within each region was measured, and the average of the buccal and palatal readings represented the relative bone density for that site.

##### Bone volume

**Fig. 6:**
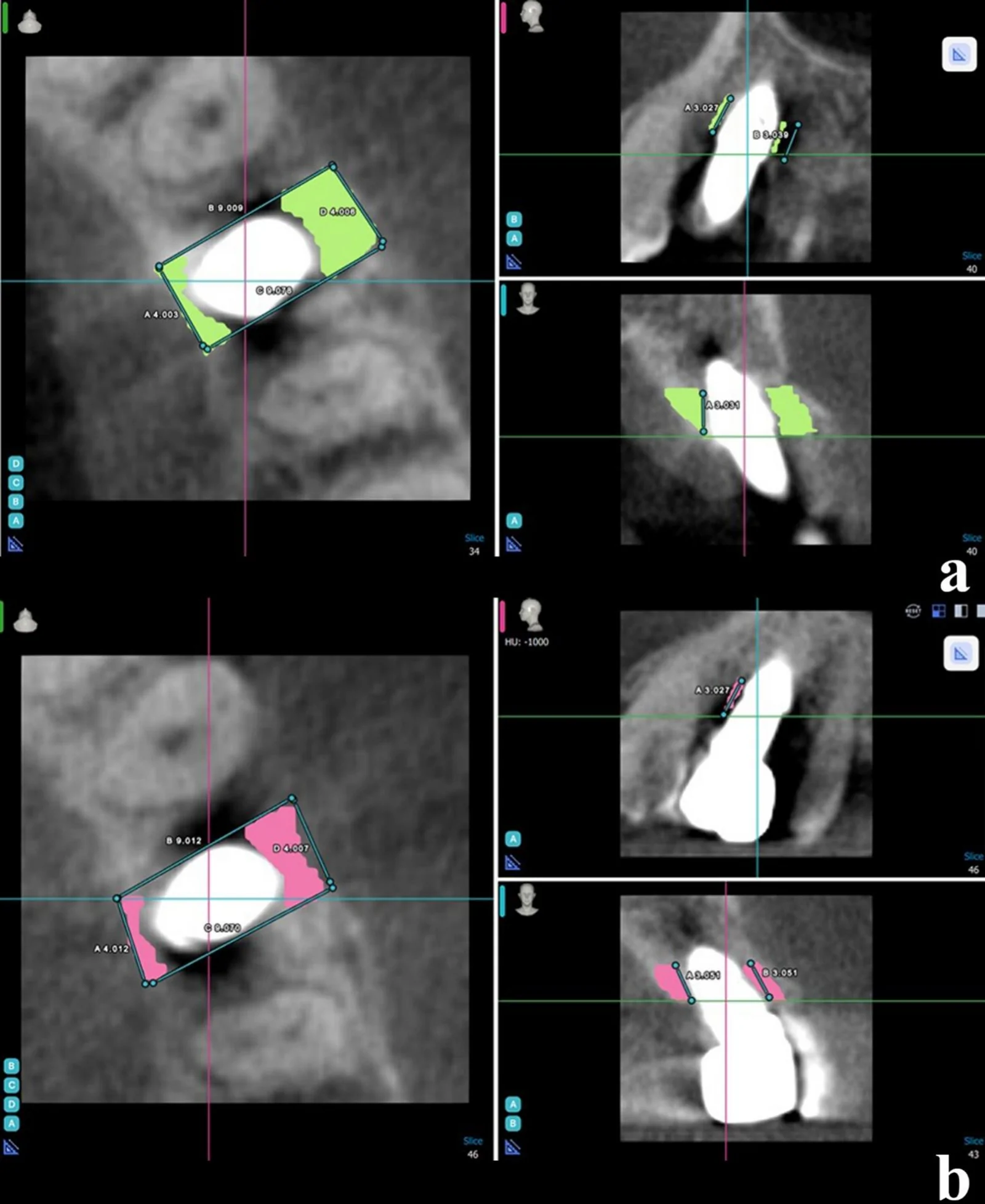
Segmentation of bone around the immediately placed implant in the study group: (**A**) Immediately after implant placement. **B** After six months, showing volumetric changes in peri-implant bone

**Fig. 7:**
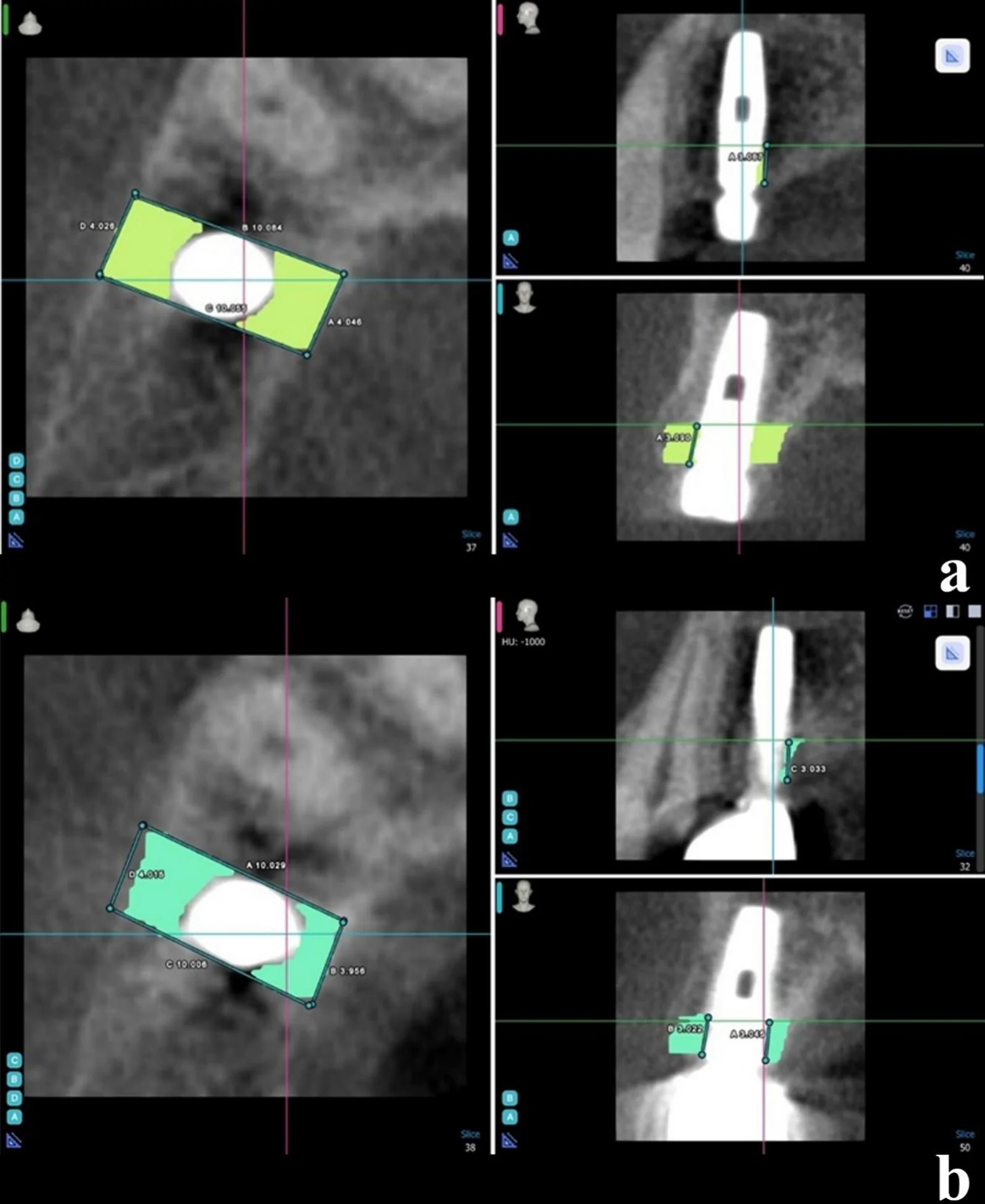
Segmentation of bone around the immediately placed implant in the control group: (**A**) Immediately after implant placement. **B** After six months, showing volumetric changes in peri-implant bone

The peri-implant bone region corresponding to the melatonin application zone (from the implant platform to 3 mm apical) was defined as the region of interest (ROI) for volumetric analysis to perform segmentation and three-dimensional reconstruction. Bone volume (mm³) was measured immediately after implant placement and after six months to assess volumetric changes within the melatonin-treated zone (Figs. 6 and 7).

##### Marginal bone level (MBL)

Measured as the vertical distance on both the mesial and distal aspects between the implant platform and the greatest coronal bone-to-implant contact. Measurements were obtained immediately after implant placement and after six months. Marginal bone loss was calculated separately for the mesial and distal aspects as the difference between the six-month and baseline measurements. The mesial and distal bone-loss values were then averaged for each implant to obtain a single implant-level marginal bone loss value, which was used for the statistical analysis.

All radiographic measurements were performed by the same calibrated examiner using identical software settings and standardized measurement protocols to minimize measurement variability.

Intra-examiner reliability was assessed using the intraclass correlation coefficient (ICC) with a 95% confidence interval. Radiographic measurements were repeated by the same examiner for 15 randomly selected implants after a 3-week interval, following the same standardized measurement protocol. The ICC values ranged from 0.942 to 0.981, demonstrating excellent intra-examiner reliability.

### Prosthetic phase

Both groups received screw-retained zirconia crowns six months after implant placement.

### Statistical analysis

Normality was tested with Shapiro–Wilk and Q–Q plots. Data were expressed as mean ± SD, median, and range. Gender was analyzed using the chi-square test. Independent *t*-test or Mann–Whitney *U* test compared groups. Repeated measures ANOVA with Bonferroni correction assessed OPG changes; paired *t*-test evaluated bone density, volume, and levels. Significance was set at *p* < 0.05. Analyses were performed using IBM SPSS version 23 (Armonk, NY, USA).

## Results

**Fig. 8:**
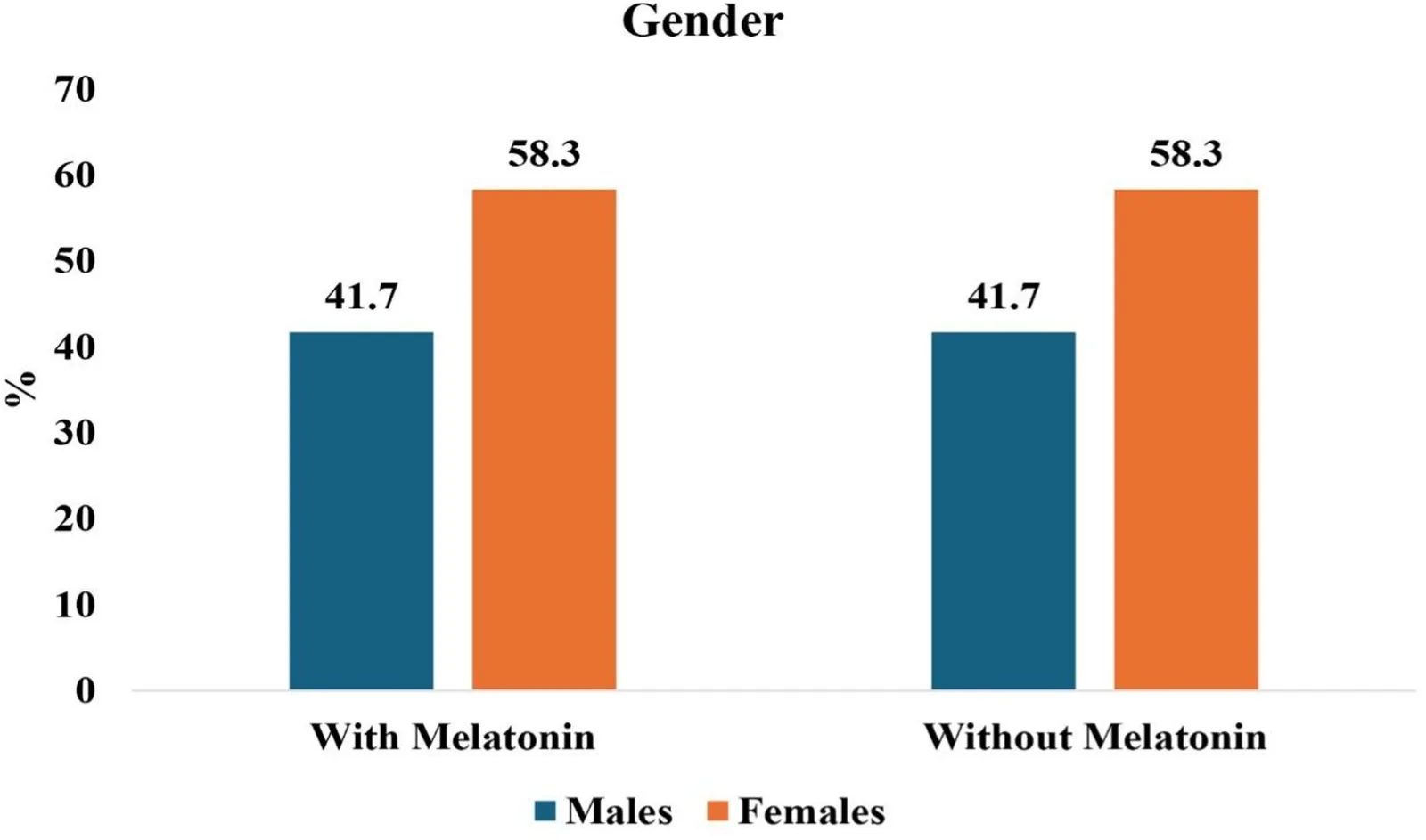
Gender distribution between the two groups

In this study, 24 participants, 10 men and 14 women, with a mean age of 38.63 years, were divided into two groups of 12: the melatonin group and the control group. The age range of the melatonin group was 28 to 49 years, with a mean age of 39.17 years, whereas the age range of the control group was 27 to 49 years, with a mean age of 38.08 years. (Table 1; Fig. 8).

**Table 1:** Demographics of the two study groups

		With Melatonin *n* = 12	Without Melatonin *n* = 12	*P* value
Age in years	Mean ± SD	39.17 ± 7.28	38.08 ± 7.29	0.719
Gender	Males	5 (41.7%)	5 (41.7%)	1.00
	Females	7 (58.3%)	7 (58.3%)	

### Osteoprotegerin level

**Fig. 9:**
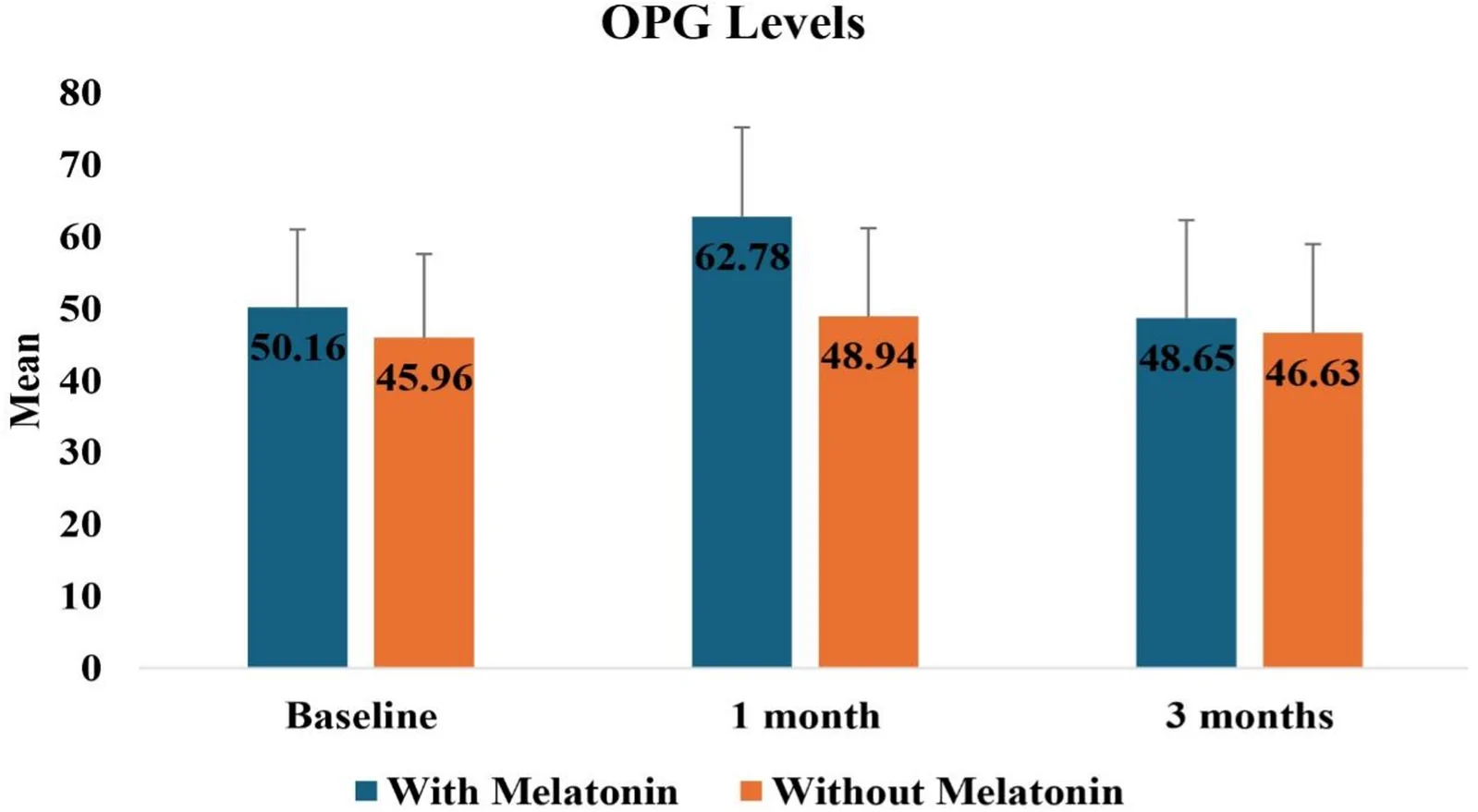
Osteoprotegerin level between patients with and without melatonin application

At baseline, there was no significant difference between groups (*p* = 0.369). After one month, the melatonin group showed significantly higher OPG levels (62.78 ± 12.39 ng/mL) than the control group (48.94 ± 12.23 ng/mL; *p* = 0.012). Within-group comparisons (*P* value²) showed significant temporal changes in both groups (melatonin: *p* = 0.002; without melatonin: *p* < 0.001). At 3 months, serum OPG levels were comparable between the two groups, with no statistically significant difference observed (*p* = 0.707), indicating that OPG levels changed significantly throughout the follow-up period (Table 2; Fig. 9).

**Table 2:** Comparison of osteoprotegerin level between patients with and without melatonin application

Follow up		With Melatonin	Without Melatonin	*P* value^a^
Baseline	Mean ± SD	50.16 ± 10.82	45.96 ± 11.62	0.369
	Median	52.55	47.85	
	Min – Max	28.90–64.20	31.40–68.60	
1 month	Mean ± SD	62.78 ± 12.39	48.94 ± 12.23	0.012*
	Median	66.45	51.65	
	Min – Max	35.40–80.40	34.10–70.00	
3 months	Mean ± SD	48.65 ± 13.62	46.63 ± 12.32	0.707
	Median	48.65	50.45	
	Min – Max	20.30–64.70	31.20–69.20	
*P* value^b^		0.002*	< 0.001*	

*Statistically significant difference at *p* value < 0.05, *P* value^a^: Independent t test, *P* value^b^: Repeated measures ANOVA test

For both groups, OPG levels increased significantly from baseline to 1 month (*p* < 0.001). A significant decline occurred from 1 month to 3 months (*p* = 0.010 with melatonin; *p* = 0.001 without melatonin) (Table 3).

**Table 3:** Pairwise comparisons of osteoprotegerin level between different follow-up intervals for patients with and without melatonin application

Follow up	Compared to	*P* value	
		With Melatonin	Without Melatonin
Baseline	1 month	< 0.001*	< 0.001*
	3 months	1.00	0.856
1 month	3 months	0.010*	0.001*

*Statistically significant difference at *p-value* < 0.05

**Fig. 10:**
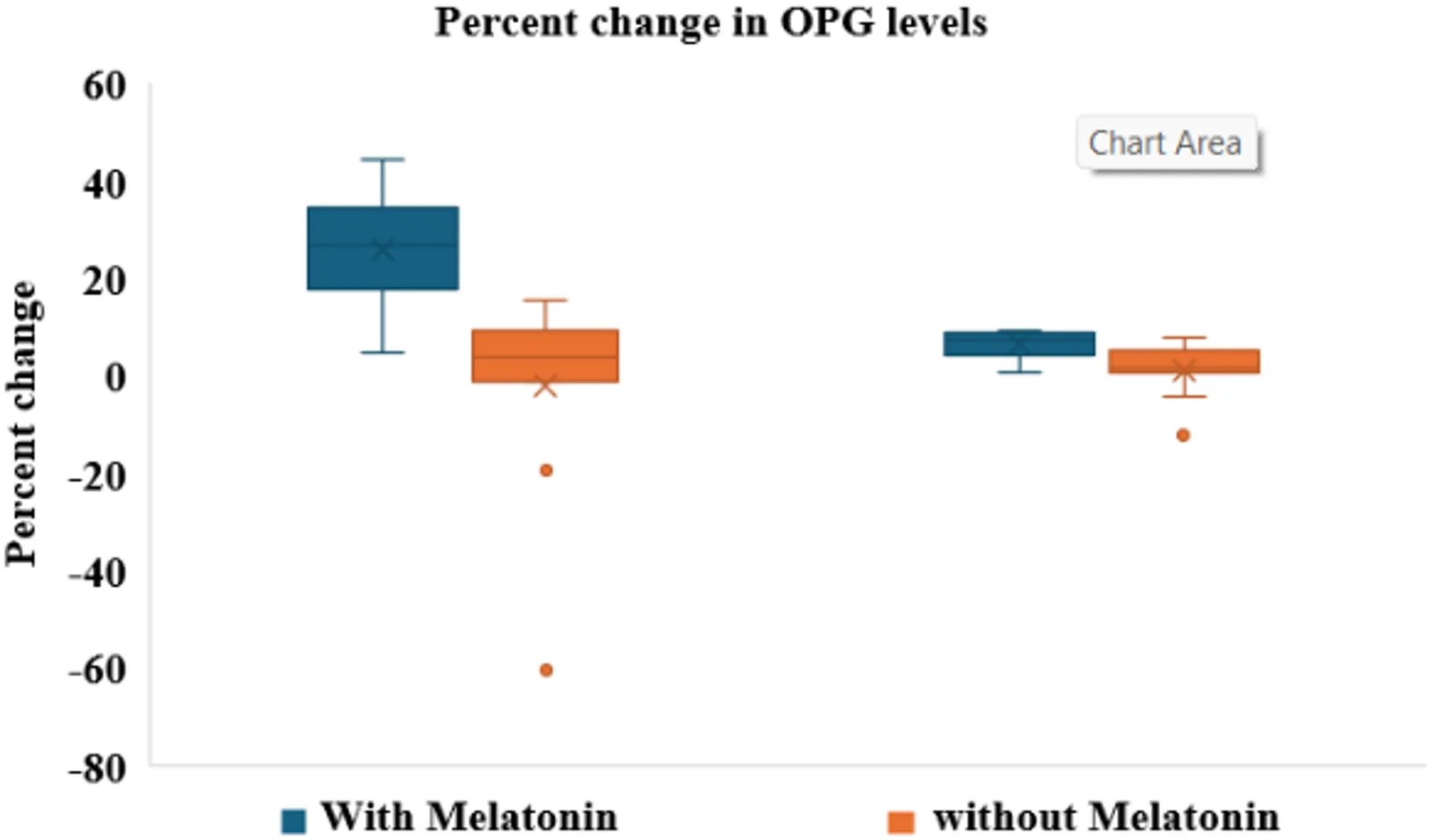
Percent change in osteoprotegerin level between patients with and without melatonin application

At 1 month, the melatonin group showed a significantly greater increase in percent change of OPG levels (mean ± SD: 26.08 ± 11.63%) compared to the control group (6.53 ± 2.84%, *p* < 0.001). At 3 months, there was no significant difference between groups (*p* = 0.729) (Table 4; Fig. 10).

**Table 4:** Comparison of percent change in osteoprotegerin level between patients with and without melatonin application

Follow up		With Melatonin	Without Melatonin	*P* value^a^
1 month	Mean ± SD	26.08 ± 11.63	6.53 ± 2.84	< 0.001*
	Median	26.84	7.58	
	Min – Max	4.79–44.32	0.85–9.41	
3 months	Mean ± SD	-2.02 ± 20.41	1.24 ± 5.24	0.729
	Median	3.94	1.69	
	Min – Max	-60.58–15.57	-12.11–7.88	

*Statistically significant difference at *p* value < 0.05, *P* value^a^: Mann Whitney U test

### Relative bone density

At baseline, patients in the melatonin group had a mean relative bone density of 457.17 ± 54.05, compared to 475.84 ± 35.22 in the control group, while there was no statistically significant difference (*p* = 0.327). After 6 months, relative bone density increased to 571.25 ± 59.59 in the melatonin group and 552.08 ± 41.91 in the control group.

**Fig. 11:**
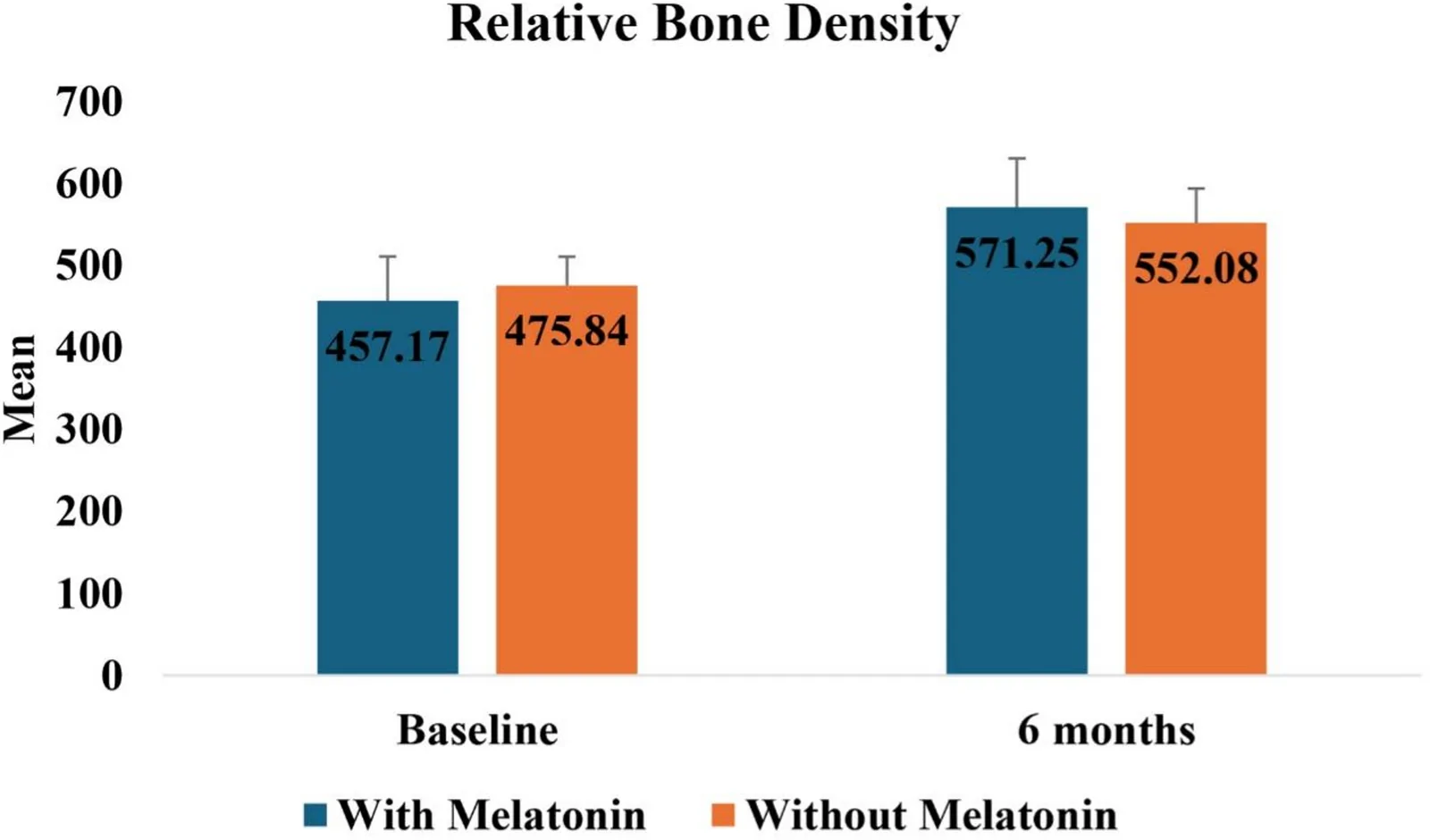
Relative bone density between the patients in the study and control groups

**Fig. 12:**
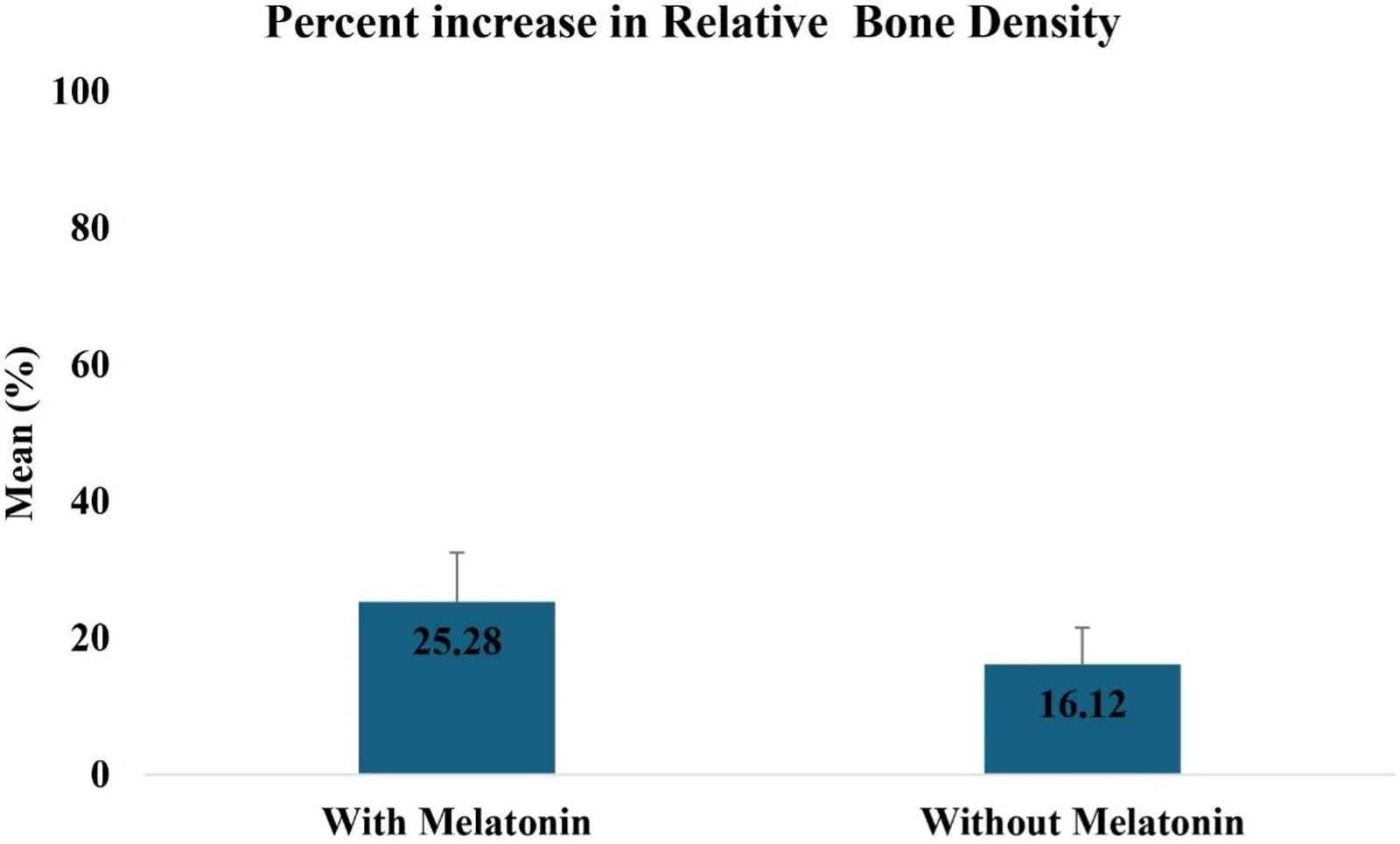
Percent increase in relative bone density

This difference at 6 months was also not statistically significant (*p* = 0.372). Both groups showed a statistically significant improvement in relative bone density over time (*p* < 0.001). The percent increase in bone density was significantly greater in the melatonin group (25.28 ± 7.22%) compared to the control group (16.12 ± 5.40%) (*p* = 0.002) (Table 5; Figs. 11 and 12).

**Table 5:** Comparison of relative bone density between study groups

Follow up		With Melatonin	Without Melatonin	*P* value^a^
Baseline	Mean ± SD	457.17 ± 54.05	475.84 ± 35.22	0.327
	Median	450.00	475.00	
	Min – Max	390.00–570.00	420.00–530.00	
6 months	Mean ± SD	571.25 ± 59.59	552.08 ± 41.91	0.372
	Median	565.00	557.50	
	Min – Max	460.00–690.00	490.00–610.00	
*P* value^b^		< 0.001*	< 0.001*	
Percent increase	Mean ± SD	25.28 ± 7.22	16.12 ± 5.40	0.002*
	Median	24.52	15.30	
	Min – Max	17.65–43.90	7.29–27.47	

*Statistically significant difference at *p* value < 0.05, *P* value^a^: Independent t test, *P* value^b^: Paired t test

### Bone volume

**Fig. 13:**
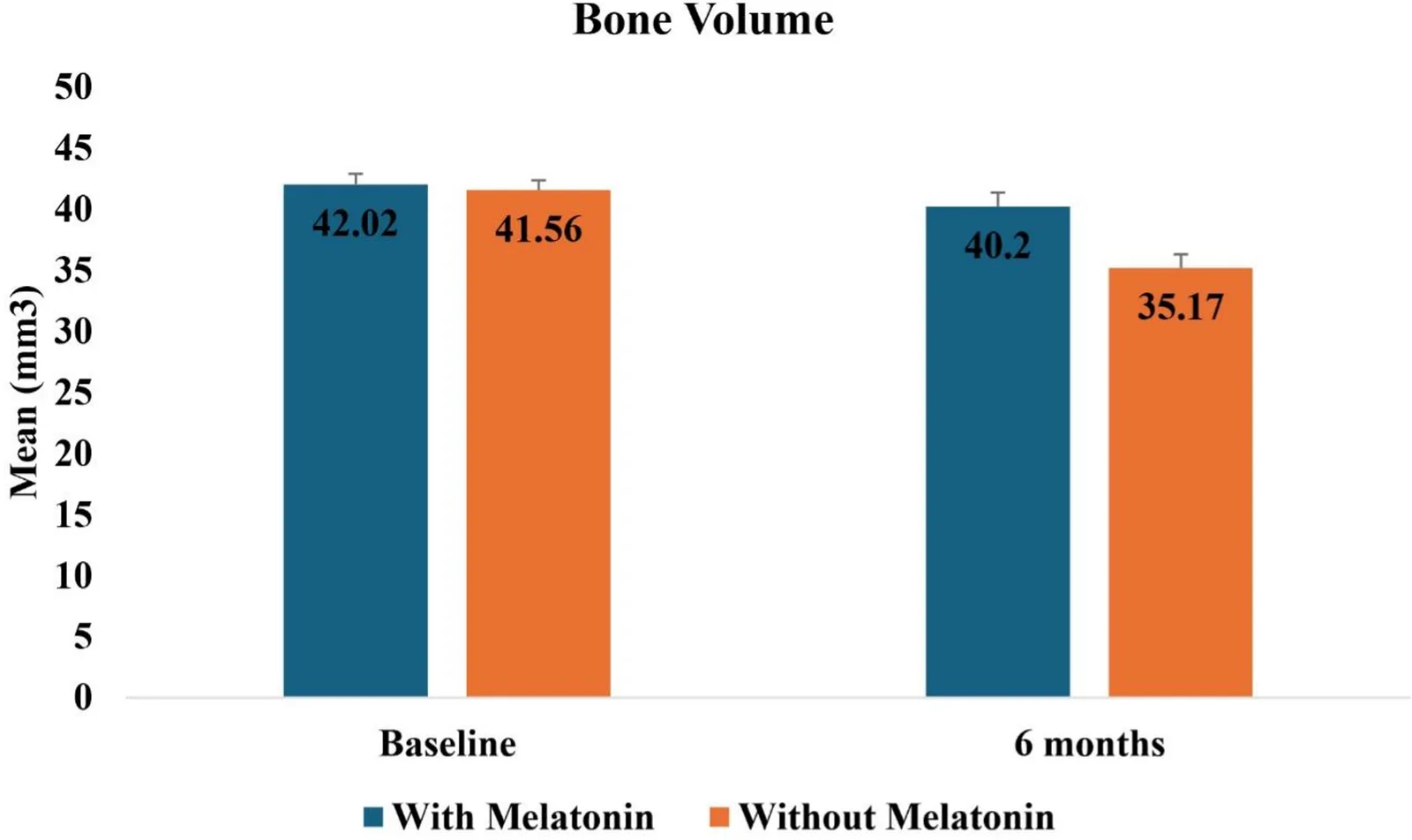
Bone volume between the patients in the study and control groups

**Fig. 14:**
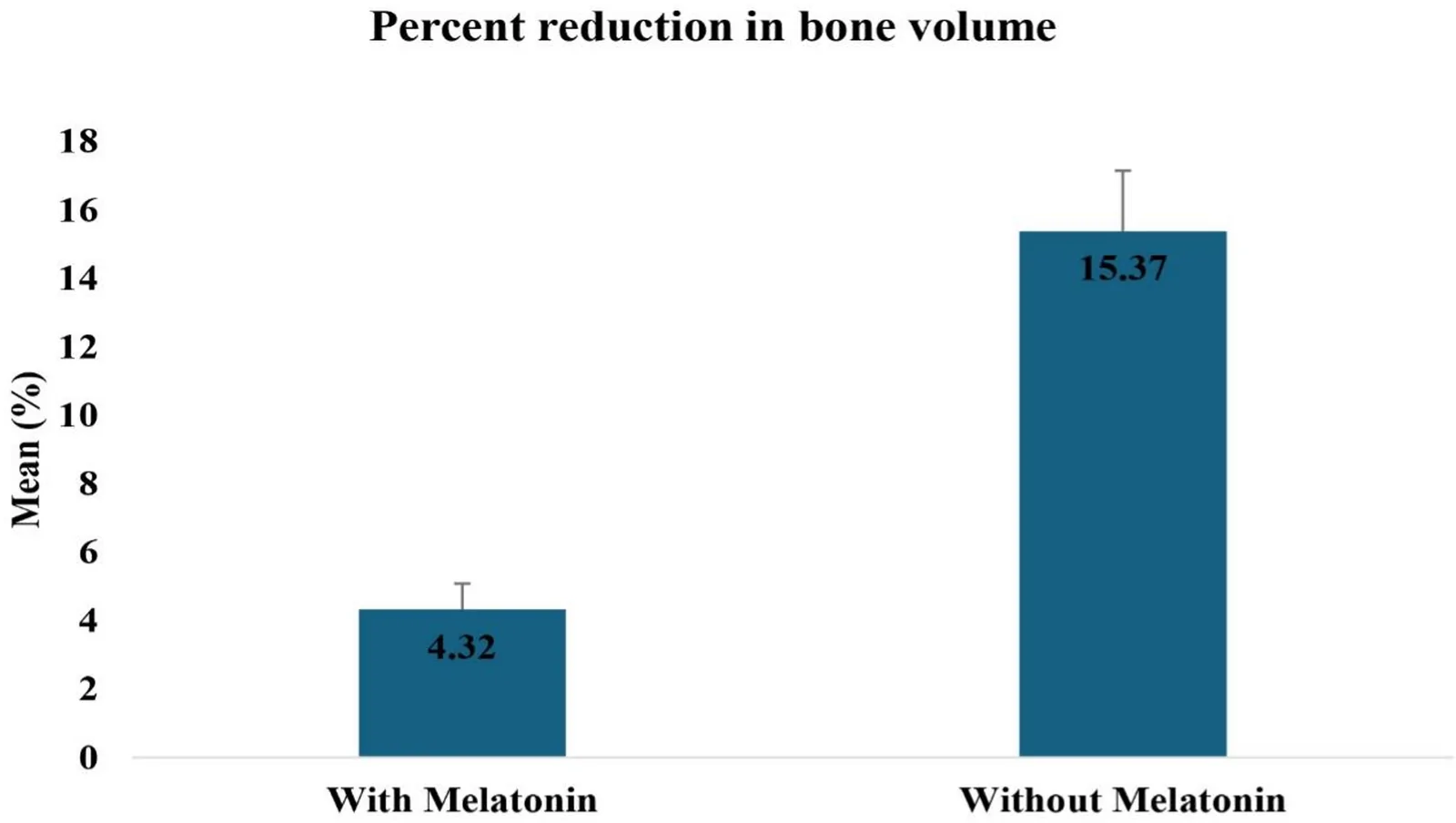
Percent reduction in bone volume

At baseline, there was no statistically significant difference in bone volume between the two groups: 42.02 ± 0.87 mm³ in the melatonin group and 41.56 ± 1.16 mm³ in the control group (*p* = 0.286). After 6 months, bone volume decreased in both groups, with a final mean of 40.20 ± 0.79 mm³ in the melatonin group and 35.17 ± 1.12 mm³ in the control group. The control group had significantly lower bone volume at 6 months compared to the melatonin group (*p* < 0.001). Both groups showed a statistically significant reduction in bone volume over time (*p* < 0.001). The percent reduction in bone volume was significantly smaller in the melatonin group (4.32 ± 0.76%) compared to the control group (15.37 ± 1.77%) (*p* < 0.001) (Table 6 Figs. 13 and 14).

**Table 6:** Comparison of bone volume (mm^3^) between study groups

Follow up		With Melatonin	Without Melatonin	*P* value^1^
Baseline	Mean ± SD	42.02 ± 0.87	41.56 ± 1.16	0.286
	Median	42.00	41.50	
	Min – Max	40.70–43.30	39.60–43.50	
6 months	Mean ± SD	40.20 ± 0.79	35.17 ± 1.12	< 0.001*
	Median	40.40	35.05	
	Min – Max	38.70–41.20	33.50–37.20	
*P* value^2^		< 0.001*	< 0.001*	
Percent reduction	Mean ± SD	4.32 ± 0.76	15.37 ± 1.77	< 0.001*
	Median	4.20	15.95	
	Min – Max	3.34–5.77	12.62–17.62	

*Statistically significant difference at *p* value < 0.05, *P* value^a^: Independent t test, *P* value^b^: Paired t test

### Marginal bone loss

**Fig. 15:**
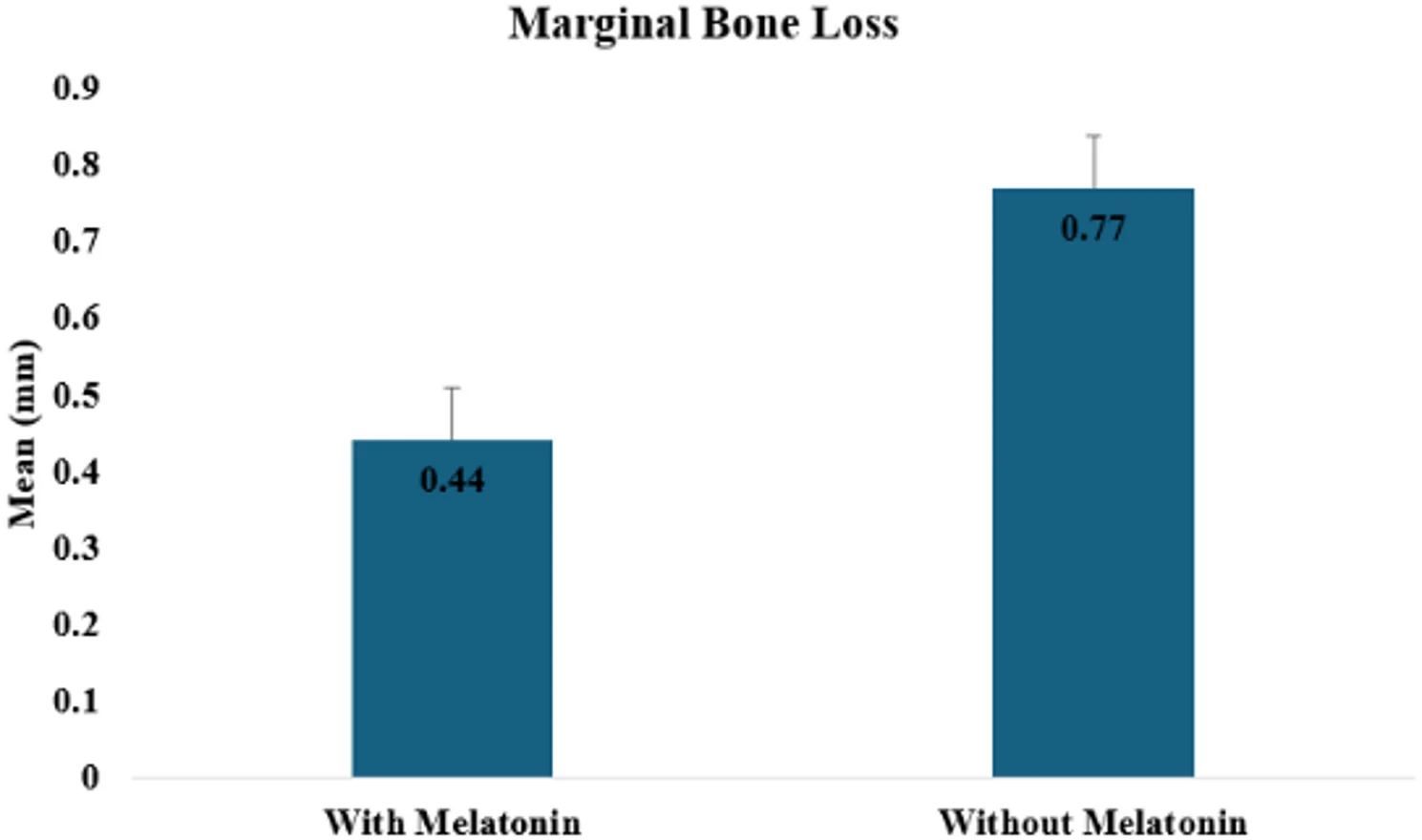
Marginal Bone loss in the study and control groups

The vertical distance between the implant platform and the most coronal bone-to-implant contact was designated as the marginal bone level. Marginal bone loss was calculated as the difference between measurements obtained at 6 months and baseline. At 6 months, the melatonin group demonstrated significantly lower marginal bone loss (0.44 ± 0.07 mm) compared with the control group (0.77 ± 0.07 mm). This difference between groups was statistically significant (independent samples t-test, *p* < 0.001) (Table 7; Fig. 15).

**Table 7:** Comparison of marginal bone loss (mm) between study groups

Follow up		With Melatonin	Without Melatonin	*P* value
Marginal bone loss after 6 months	Mean ± SD	0.44 ± 0.07	0.77 ± 0.07	< 0.001*
	Median	0.45	0.75	
	Min – Max	0.30–0.55	0.70–0.90	

*Statistically significant difference at *p* value < 0.05, *P* value: Independent t-test

## Discussion

The present randomized controlled clinical trial evaluated the biological and radiographic effects of locally applied melatonin around immediately placed implants in the maxillary premolar area. Local melatonin application was associated with a more favourable peri-implant healing response than immediate implant placement alone, greater preservation of peri-implant bone volume, a significantly greater percentage increase in relative bone density from baseline, and reduced marginal bone loss over the six-month observation period, alongside higher circulating OPG levels as a supportive biological finding. These findings suggest a possible role for locally delivered melatonin as a bioactive adjunct in modulating peri-implant bone remodelling following immediate implant placement.

### Biochemical findings

The favourable biochemical findings observed in the present study may be explained by the well-established role of melatonin in regulating early bone remodelling. Melatonin enhances osteoblastic activity while suppressing osteoclastogenesis through modulation of the RANK/RANKL/OPG signalling pathway, thereby promoting bone formation and reducing bone resorption. Moreover, its antioxidant and anti-inflammatory properties may further support a biological environment favourable for peri-implant healing. These biological mechanisms have been consistently demonstrated in both experimental and clinical studies investigating the osteogenic effects of melatonin [9, 29, 30]. This interpretation is also supported by experimental evidence showing that local application of 3 mg melatonin powder improved biological implant stability, although no statistically significant difference was observed in removal torque between melatonin-treated and control implants [31]. 

The present findings are in agreement with those of Refahee et al. [28], who reported a significant increase in serum OPG levels following local melatonin application after impacted mandibular third molar surgery, suggesting that the osteogenic effects of locally delivered melatonin are not limited to implant therapy but may also extend to other oral surgical procedures involving bone healing. Although the biological environment surrounding an extraction socket differs from that of an immediately placed implant, both studies support the concept that local melatonin administration promotes a bone remodelling profile favouring bone formation. Importantly, the biochemical findings should not be interpreted in isolation. Unlike previous clinical investigations that primarily evaluated either biochemical or radiographic parameters, the present randomized controlled trial integrated longitudinal serum OPG assessment with three complementary radiographic outcome measures, namely peri-implant bone volume, relative bone density, and marginal bone loss. The parallel improvement observed in the biochemical and radiographic findings may support the proposed biological effects of locally applied melatonin on peri-implant bone remodelling. However, because OPG was assessed as a secondary exploratory outcome, these findings should be interpreted with caution and cannot establish a causal biological mechanism. Nevertheless, because melatonin was locally applied around the implant, peri-implant crevicular fluid (PICF) analysis could have provided additional site-specific information regarding the biological events occurring at the implant–bone interface. Previous studies have highlighted the value of PICF as a reliable biological medium for monitoring peri-implant biomarkers during the healing process [32, 33].

### Radiographic findings

The favourable radiographic outcomes observed in the present study further support the proposed biological effects of locally delivered melatonin on peri-implant bone remodelling.

The significantly greater preservation of peri-implant bone volume, together with the significantly greater percentage increase in relative bone density from baseline observed in the melatonin group, suggests a potential favourable effect of local melatonin administration on early peri-implant bone remodelling. However, the between-group difference in absolute relative bone density at six months was not statistically significant. These findings are biologically plausible considering the known osteogenic, antioxidant, and anti-inflammatory properties of melatonin, which collectively promote a microenvironment favourable for bone regeneration and remodelling [10, 29, 30].

Our findings are consistent with previous clinical investigations evaluating the local use of melatonin in implant dentistry. Ravi Kiran et al. [3] reported significantly greater peri-implant bone density around immediately placed implants treated with topical melatonin using CBCT assessment. Similarly, Abdel-Dayem et al. [4], Awwad et al. [34], and El-Gammal et al. [35] reported favourable radiographic outcomes following local melatonin application. Additional clinical studies evaluating melatonin alone or in combination with other biomaterials have also suggested improvements in peri-implant bone healing [36–38]. Despite differences in melatonin formulation, delivery vehicle, implant protocol, anatomical site, adjunctive biomaterials, and follow-up duration, the overall body of evidence suggests that locally applied melatonin may enhance peri-implant bone healing.

Nevertheless, an important distinction should be emphasized. Most previously published clinical studies primarily relied on conventional radiographic measurements, including bone density or marginal bone level, to evaluate peri-implant healing. In contrast, the present randomized controlled trial incorporated three-dimensional volumetric assessment of peri-implant bone in addition to relative bone density and marginal bone loss. This comprehensive radiographic evaluation provides a more detailed characterization of peri-implant bone remodelling than conventional two-dimensional assessments and represents a methodological advantage of the present study. Moreover, the radiographic findings were accompanied by favourable biochemical changes, as the increase in serum OPG levels paralleled the structural changes observed on CBCT. The consistency between these complementary outcome measures provides additional support for the proposed biological effects of locally applied melatonin.

Preservation of peri-implant crestal bone is one of the key determinants of long-term implant success, as excessive early bone remodelling may adversely affect both osseointegration and peri-implant tissue stability. The significantly lower marginal bone loss observed in the present study suggests that locally applied melatonin favourably modulates early peri-implant bone remodelling rather than merely accelerating new bone formation. This observation is consistent with the known osteogenic and antiresorptive properties of melatonin, which regulate bone remodelling through modulation of the RANK/RANKL/OPG pathway while reducing oxidative stress and inflammation [7, 8, 28, 29].

The present findings are consistent with those of El-Gammal et al. [35], Awwad et al. [34], Hazzaa et al. [39], and Abdel-Aziz et al. [37], all of whom reported improved peri-implant bone preservation following local melatonin application. However, Ravi Kiran et al. [3] did not detect a significant reduction in crestal bone loss despite observing higher peri-implant bone density. This discrepancy may reflect differences in study design, sample size, follow-up duration, melatonin delivery protocols, and radiographic assessment methods rather than true biological inconsistency. Recently, Stacchi et al. [40] emphasized in an international clinical consensus that early peri-implant bone remodelling is a multifactorial biological process influenced by anatomical, surgical, implant-, prosthetic-, and patient-related factors. Accordingly, the interpretation of interventions intended to modulate peri-implant bone remodelling requires careful control of potential confounding variables. In the present study, several of these variables were standardized through strict case selection and a uniform surgical protocol, thereby reducing potential sources of variability. Nevertheless, factors such as peri-implant soft tissue phenotype and other patient-related biological variables were beyond the scope of the present investigation and should be considered in future randomized clinical trials [41]. In addition, future studies incorporating a placebo-vehicle control group would allow more definitive isolation of the biological effects of melatonin from any potential transient physical contribution of the applied mixture, and are recommended to further substantiate the mechanism underlying the observed outcomes.

Taken together, the findings of the present study suggest that the biological effects of locally applied melatonin should not be interpreted based on a single outcome measure. Rather, the parallel improvement in serum OPG levels together with the preservation of peri-implant bone volume, a significantly greater percentage increase in relative bone density from baseline and reduced marginal bone loss provides a more comprehensive assessment of peri-implant bone remodelling. This integrated evaluation may also help explain the variability among previous clinical studies, many of which relied on a single biochemical, clinical, or radiographic outcome. Consequently, the present findings add to the existing clinical evidence supporting the biological rationale for local melatonin application during immediate implant placement and highlight the value of integrating complementary biochemical and radiographic outcome measures in future clinical research.

### Limitations

The present study has several limitations that should be considered when interpreting the findings. First, the relatively small sample size, six-month follow-up period, and restriction to immediately placed implants in maxillary premolars limit the generalizability of the findings; the six-month follow-up in particular reflects only the early phase of peri-implant bone healing and does not permit conclusions regarding long-term bone remodelling. Second, although relative bone density was assessed using standardized CBCT imaging, the influence of beam-hardening and metallic artefacts inherent to implant imaging cannot be completely excluded. Third, several anatomical, prosthetic, and clinical parameters known to influence peri-implant outcomes were not evaluated, including supra-crestal mucosal thickness, emergence profile and angle, implant stability quotient (ISQ), probing depth, bleeding on probing, and peri-implant soft tissue conditions. Fourth, while the local melatonin application protocol was standardized by complete filling of the peri-implant defect, the exact amount of melatonin retained at each site could not be quantified due to minor inter-patient variation in defect dimensions. In addition, the melatonin–saline mixture applied to the peri-implant gap lacked the structural properties (rigidity, particulate architecture, and in vivo persistence) associated with established space-maintaining biomaterials, a transient physical contribution to the peri-implant space in the immediate period following application cannot be entirely excluded, and this possibility should be considered when interpreting the reported bone volume outcomes. Finally, OPG was evaluated in serum rather than in peri-implant crevicular fluid (PICF), which may have limited assessment of site-specific biological events at the implant–bone interface, and RANKL was not assessed; as OPG was a predefined secondary/exploratory outcome intended to complement the primary radiographic findings, these results should be interpreted with appropriate caution. Future randomized controlled trials incorporating these anatomical, prosthetic, and clinical parameters, standardized local drug delivery quantification, longer post-loading follow-up, and combined serum/PICF biomarker analysis (including OPG and RANKL) are warranted to more comprehensively characterize the local and systemic mechanisms underlying peri-implant bone remodelling following local melatonin application.

## Conclusion

Within the limitations of this randomized controlled clinical trial, local application of melatonin around immediately placed implants was associated with favourable radiographic outcomes during early healing, including greater preservation of peri-implant bone volume, a significantly greater percentage increase in relative bone density from baseline, and reduced marginal bone loss compared with the control group. These radiographic findings were accompanied by an early increase in serum OPG levels, a secondary biochemical outcome that provides complementary biological evidence alongside the radiographic findings. Collectively, these findings suggest that locally applied melatonin may enhance early peri-implant bone healing following immediate implant placement during the six-month follow-up period.

Nevertheless, larger randomized controlled trials with longer follow-up periods, adequately powered for biochemical as well as radiographic outcomes, and incorporating comprehensive assessment of both local and systemic biomarkers are required to confirm the long-term clinical effectiveness of this approach and to better define its role in routine implant therapy.

## Supplementary Information

Supplementary Material 1.

## Data Availability

Availability of data and materialsAll data generated or analyzed during this study are included in this published article [and its supplementary information files].

## Abbreviations

OPG: Osteoprotegerin
RANK: Receptor activator of nuclear factor kappa-B
RANKL: Receptor activator of nuclear factor kappa-B ligand
CBCT: Cone beam computed tomography
GIT: Gastrointestinal tract
COX-2: Cyclooxygenase-2
SD: Standard deviation
SPSS: Statistical package for social sciences
Ml: Milli-Liter
Mg: Milligram
Mm^3^: Millimeter cubic
^O^C: Degree celsius
G: Gravity
ELISA: Enzyme-linked immunosorbent assay
HRP: Horseradish peroxidase
N·cm: Newton centimetre
RPM: Revolutions per minute
mRNA: messenger ribonucleic acid
MT1: Melatonin receptor type 1
MT2: Melatonin receptor type 2
ABG: Autogenous bone graft
PICF: Peri-implant crevicular fluid
ISQ: Implant stability quotient

## References

[1] Ravella H, Rao M, Manthena S, Koneru S, Preethi P. The Effect of Melatonin on Implant Stability and Marginal Bone Level around The Implant. J Dent Indones. 2022;29:105–12. 10.14693/jdi.v29i2.1319.

[2] n der Weijden F, Dell’Acqua F, Slot DE. Alveolar bone dimensional changes of post-extraction sockets in humans: a systematic review. J Clin Periodontol. 2009;36:1048–58. 10.1111/j.1600-051X.2009.01482.x. 19929956

[3] Ravi Kiran S, Bammidi N, Kumar AK, Kumar PS, Karnam Y. Evaluation of the Effect of Topical Melatonin Application on Immediately Placed Dental Implants Using Cone Beam Computed Tomography (CBCT). Cureus. 2022;14:e25233. 10.7759/cureus.25233. PMC9217677 35755538

[4] Abdel-Dayem H, Abdel-Alim HM, Banasr FH. Topical application of melatonin around immediate implants. Am J Res Commun. 2014;2:1–12.

[5] Bagherifard A, Hosseinzadeh A, Koosha F, Sheibani M, Karimi-Behnagh A, Reiter RJ, et al. Melatonin and bone-related diseases: an updated mechanistic overview of current evidence and future prospects. Osteoporos Int. 2023;34(10):1677–701. 10.1007/s00198-023-06836-1. 37393580

[6] Tresguerres IF, Clemente C, Blanco L, Khraisat A, Tamimi F, Tresguerres JA. Effects of local melatonin application on implant osseointegration. Clin Implant Dent Relat Res. 2012;14:395–9. 10.1111/j.1708-8208.2010.00271.x. 20455901

[7] Atilgan M, Arpag OF, Ozcan O, Kacmaz F. Biochemical evaluation of the effects of concentrated growth factor liquid on osseointegration: a split-mouth design study. Int J Oral Maxillofac Implants. 2023;38(6):1182–90. 10.11607/jomi.10066. 38085750

[8] Udagawa N, Koide M, Nakamura M, Nakamichi Y, Yamashita T, Uehara S, et al. Osteoclast differentiation by RANKL and OPG signaling pathways. J Bone Min Metab. 2021;39:19–26. 10.1007/s00774-020-01162-6. 33079279

[9] Tian Y, Ming J. Melatonin inhibits osteoclastogenesis via RANKL/OPG suppression mediated by Rev-Erbα in osteoblasts. J Cell Mol Med. 2022;26:4032–47. 10.1111/jcmm.17440. PMC9279587 35726597

[10] Maria S, Witt-Enderby PA. Melatonin effects on bone: potential use for the prevention and treatment for osteopenia, osteoporosis, and periodontal disease and for use in bone-grafting procedures. J Pineal Res. 2014;56:115–25. 10.1111/jpi.12116. 24372640

[11] Clafshenkel WP, Rutkowski JL, Palchesko RN, Romeo JD, McGowan KA, Gawalt ES, et al. A novel calcium aluminate-melatonin scaffold enhances bone regeneration within a calvarial defect. J Pineal Res. 2012;53:206–18. 10.1111/j.1600-079X.2012.00989.x. 22462771

[12] Guardia J, Gómez-Moreno G, Ferrera MJ, Cutando A. Evaluation of effects of topic melatonin on implant surface at 5 and 8 weeks in Beagle dogs. Clin Implant Dent Relat Res. 2011;13:262–8. 10.1111/j.1708-8208.2009.00211.x. 19681939

[13] Acikan I, Mehmet G, Artas G, Yaman F, Deniz G, Bulmus O, et al. Systemic melatonin application increases bone formation in mandibular distraction osteogenesis. Braz Oral Res. 2018;32:e85. 10.1590/1807-3107bor-2018.vol32.0085. 30281683

[14] Zhou MS, Tao ZS. Systemic administration with melatonin in the daytime has a better effect on promoting osseointegration of titanium rods in ovariectomized rats. Bone Joint Res. 2022;11:751–62. 10.1302/2046-3758.1111.Bjr-2022-0017.R2. PMC9680200 36317318

[15] Gómez-Moreno G, Aguilar-Salvatierra A, Boquete-Castro A, Guardia J, Piattelli A, Perrotti V, et al. Outcomes of topical applications of melatonin in implant dentistry: A systematic review. Implant Dent. 2015;24(1):25–30. 10.1097/ID.0000000000000186. 25621548

[16] Oliveira PHC, Lemos CAA, Cantiga-Silva C, Faria FD, Cintra LTA, Pellizzer EP. Melatonin as an adjunctive treatment on dental procedures: A systematic review. Oral Dis. 2022;28(7):1770–82. 10.1111/odi.13941. 34152659

[17] ElSholkamy M, Makawi DM, ElKhateeb SM, Soliman T, Soliman RA. Clinical, radiographic and histomorphometric assessment of the effects of melatonin gel mixed with a xenograft in augmentation of the maxillary sinus: a randomized controlled clinical trial. BMC Oral Health. 2026;26:732. 10.1186/s12903-026-08201-0. PMC13110429 41987188

[18] Pratap AJ, Srinath R, Praveen NC, Sustarwar P, Sadarjoshi M, Almalki SA, et al. Evaluation of melatonin gel as local drug delivery system for the treatment of periodontitis: a split-mouth randomized controlled trial. BMC Oral Health. 2025;25:230. 10.1186/s12903-025-05598-y. PMC11827357 39948592

[19] Liu RY, Li L, Zhang ZT, Wu T, Lin S, Zhang XT. Clinical efficacy of melatonin as adjunctive therapy to non-surgical treatment of periodontitis: A systematic review and meta-analysis. Inflammopharmacology. 2022;30(3):695–704. 10.1007/s10787-022-00959-3. 35290552

[20] Schulz KF, Altman DG, Moher D. CONSORT 2010 statement: updated guidelines for reporting parallel group randomised trials. BMJ. 2010;340:c332. 10.1136/bmj.c332. PMC2844940 20332509

[21] World Medical Association. World Medical Association Declaration of Helsinki: Ethical Principles for Medical Research Involving Human Participants. JAMA. 2025;333(1):71–4. 10.1001/jama.2024.21972. 39425955

[22] Hatem A, Tawfik M, Mowafey B, elgammal M, Salem AS. Melatonin Application with Indirect Maxillary Sinus Lift and Simultaneous Implant Placement: A Promising New Approach. Egypt Dent J. 2022;68:3105–15. 10.21608/edj.2022.140272.2121.

[23] Rosner B. Fundamentals of biostatistics. 7th ed. Boston: Brooks/Cole; 2015. pp. 269–301.

[24] Brant R. Power/Sample Size Calculator: Inference for Means: Comparing Two Independent Samples [Internet]. Vancouver (BC): University of British Columbia; Available from: https://www.stat.ubc.ca/~rollin/stats/ssize/n2. Accessed 13 Nov 2023.

[25] Tonetti MS, Jung RE, Avila-Ortiz G, Blanco J, Cosyn J, Fickl S, et al. Management of the extraction socket and timing of implant placement: Consensus report and clinical recommendations of Group 3 of the XV European Workshop in Periodontology. J Clin Periodontol. 2019;46(Suppl 21):183–94. 10.1111/jcpe.13131. 31215112

[26] Mustakim KR, Eo MY, Lee JY, Myoung H, Seo MH, Kim SM. Guidance and rationale for the immediate implant placement in the maxillary molar. J Korean Assoc Oral Maxillofac Surg. 2023;49:30–42. 10.5125/jkaoms.2023.49.1.30. PMC9985995 36859373

[27] Sealed Envelope Ltd. Simple randomisation service. London: Sealed Envelope Ltd. 2022. Available from: https://www.sealedenvelope.com/simple-randomiser/v1/. Accessed 13 Nov 2023.

[28] Refahee SM, Aboulmagd I, Ragab R, Abdel Aziz O, Ahmed W, Shabaan AA. The Effect of Local Melatonin Application Following the Removal of an Impacted Mandibular Third Molar. J Oral Maxillofac Surg. 2023;81:622–31. 10.1016/j.joms.2023.01.010. 36796435

[29] Malakoti F, Zare F, Zarezadeh R, Raei Sadigh A, Sadeghpour A, Majidinia M, et al. The role of melatonin in bone regeneration: A review of involved signaling pathways. Biochimie. 2022;202:56–70. 10.1016/j.biochi.2022.08.008. 36007758

[30] Kim SS, Jeong SP, Park BS, Kim IR. Melatonin attenuates RANKL-induced osteoclastogenesis via inhibition of Atp6v0d2 and DC-STAMP through MAPK and NFATc1 signaling pathways. Molecules. 2022;27(2):501. 10.3390/molecules27020501. PMC8781594 35056817

[31] Marquinez Garcia PR, Ibañez JC, Grenón MS. Local Application of Melatonin on Dental Implant Osseointegration: Analysis of Removal Torque. Experimental Study on Rabbits. Eur J Dent Oral Health. 2023;4(5):16–24. 10.24018/ejdent.2023.4.5.283.

[32] Sayardoust S, Omar O, Thomsen P. Gene expression in peri-implant crevicular fluid of smokers and nonsmokers. 1. The early phase of osseointegration. Clin Implant Dent Relat Res. 2017;19(4):681–93. 10.1111/cid.12486. 28470893

[33] Dogan SB, Kurtiş B, Tüter G, Serdar M, Watanabe K, Karakış S. Evaluation of clinical parameters and levels of proinflammatory cytokines in the crevicular fluid around dental implants in patients with type 2 diabetes mellitus. Int J Oral Maxillofac Implants. 2015;30(5):1119–27. 10.11607/jomi.3787. 26394349

[34] Awwad YM, Khalil MA, Al Sayed SA. Effect of melatonin application around immediate implants. Al-Azhar J Dent Sci. 2019;22:187–93.

[35] El-Gammal MY, Salem AS, Anees MM, Tawfik MA. Clinical and Radiographic Evaluation of Immediate Loaded Dental Implants With Local Application of Melatonin: A Preliminary Randomized Controlled Clinical Trial. J Oral Implantol. 2016;42:119–25. 10.1563/aaid-joi-D-14-00277. 26103559

[36] Nasr A, Shoeib M, Darhous M, El Barbary A. The Effect of Melatonin and Hyaluronic Acid Mixture on Hard Tissue Dimensional Changes Around Immediate Post-Extraction Implants. Egypt Dent J. 2022;68:565–77. 10.21608/edj.2022.110319.1898.

[37] Abdel-Aziz L, Ahmed MIR. Evaluation of local delivered platelet–rich fibrin versus Melatonin gel on the osseointegration of dental implant: a randomized controlled clinical trial. Tanta Dent J. 2023;20:47–53. 10.4103/tdj.tdj_54_22.

[38] Amer S. Management Of Gap Distance Around Immediate Implants With Topical Melatonin Gel and Hyaluronic Acid (A Randomized Clinical Trial). Egypt Dent J. 2022;68:2209–22. 10.21608/edj.2022.125959.2012.

[39] Hazzaa HHA, El-Kilani NS, Elsayed SA, Abd El Massieh PM. Evaluation of Immediate Implants Augmented with Autogenous Bone/Melatonin Composite Graft in the Esthetic Zone: A Randomized Controlled Trial. J Prosthodont. 2019;28:e637–42. 10.1111/jopr.12631. 29143462

[40] Stacchi C, Testori T, Rapani A, Arai Y, Avila-Ortiz G, Blanco-Carrión J, et al. Prevention of initial bone remodelling around dental implants: An international clinical consensus utilising the Delphi method. Int J Oral Implantol (Berl). 2026;19(1):61–74. 41823005

[41] Insua A, Galindo-Moreno P, Miron RJ, Wang HL, Monje A. Emerging factors affecting peri-implant bone metabolism. Periodontol 2000. 2024;94:27–78. 10.1111/prd.12532. 37904311

